# Superior Mesenteric Venous Thrombosis after Laparoscopic Exploration for Small Bowel Obstruction

**DOI:** 10.1155/2013/952383

**Published:** 2013-12-22

**Authors:** Hideki Katagiri, Shozo Kunizaki, Mayu Shimaguchi, Yasuo Yoshinaga, Yukihiro Kanda, Alan T. Lefor, Ken Mizokami

**Affiliations:** ^1^Department of Surgery, Tokyo Bay Urayasu Ichikawa Medical Center (Noguchi Hideyo Memorial International Hospital), 3-4-34 Todaijima, Urayasu, Chiba 279-0001, Japan; ^2^Department of Surgery, Jichi Medical University, 1-3311 Yakushiji, Shimotsuke, Tochigi 329-0498, Japan

## Abstract

Mesenteric venous thrombosis is a rare cause of intestinal ischemia which is potentially life-threatening because it can lead to intestinal infarction. Mesenteric venous thrombosis rarely develops after abdominal surgery and is usually associated with coagulation disorders. Associated symptoms are generally subtle or nonspecific, often resulting in delayed diagnosis. A 68-year-old woman underwent laparoscopic exploration for small bowel obstruction, secondary to adhesions. During the procedure, an intestinal perforation was identified and repaired. Postoperatively, the abdominal pain persisted and repeat exploration was undertaken. At repeat exploration, a perforation was identified in the small bowel with a surrounding abscess. After the second operation, the abdominal pain improved but anorexia persisted. Contrast enhanced abdominal computed tomography was performed which revealed superior mesenteric venous thrombosis. Anticoagulation therapy with heparin was started immediately and the thrombus resolved over the next 6 days. Although rare, this complication must be considered in patients after abdominal surgery with unexplained abdominal symptoms.

## 1. Introduction

Mesenteric venous thrombosis is an unusual cause of intestinal ischemia and potentially life-threatening because it can result in intestinal infarction. Mesenteric venous thrombosis accounts for 5 to 15% of all mesenteric ischemic events and usually involves the superior mesenteric vein [1–4]. Several cases of mesenteric venous thrombosis after abdominal surgery have been reported; however, mesenteric venous thrombosis after surgery for abdominal sepsis is especially uncommon. We report a case of superior mesenteric venous thrombosis after abdominal abscess with small intestinal perforation, successfully treated by systemic anticoagulation therapy.

## 2. Case Presentation

A 68-year-old woman with a history of previous abdominal surgery presented with abdominal pain and vomiting. One day prior to admission, she noted the gradual onset of abdominal pain. She had one bowel movement but the abdominal pain persisted. The pain was intermittent and gradually worsened. She vomited several times. She underwent a hernia repair 15 years previously and had a lower midline incision, although the details of that procedure were unavailable. On physical examination, her lower abdomen was slightly distended with mild tenderness to palpation. Dilated intestine was palpable, but there were no signs of peritonitis. Nasogastric suction was initiated but inadequate and the abdominal pain persisted. Abdominal CT scan revealed dilated loops of small intestine with a small amount of ascites.

The diagnosis of small bowel obstruction was established and exploration undertaken. This was begun laparoscopically which demonstrated multiple areas of adherent loops of small bowel. The adhesions were lysed sharply and further exploration revealed a small bowel perforation, which was repaired in a conventional manner after conversion to open laparotomy.

On postoperative day (POD) 1, her temperature increased to 39°C; however, it resolved over five days without specific treatment. The abdominal pain persisted and became more intense on POD 6. Abdominal CT scan was performed on POD 7, which showed a small fluid collection with some air. Due to persistence and increasing severity of the abdominal pain, repeat operative exploration was undertaken on POD 8. Exploration revealed abscesses in the abdominal wall and between loops of small bowel, as well as a site of perforation, which was resected and repaired with a primary anastomosis.

**Figure 1:**
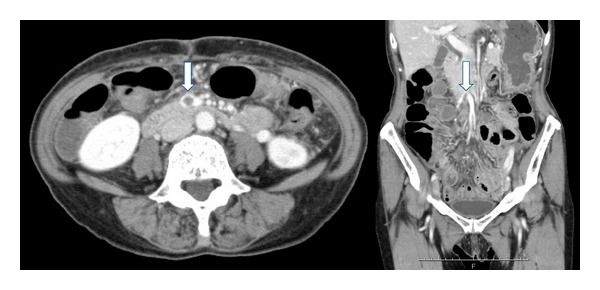
Contrast enhanced computed tomographic scan (axial and coronal views) of the abdomen demonstrated a filling defect in the superior mesenteric vein (arrow), suggesting thrombus.

**Figure 2:**
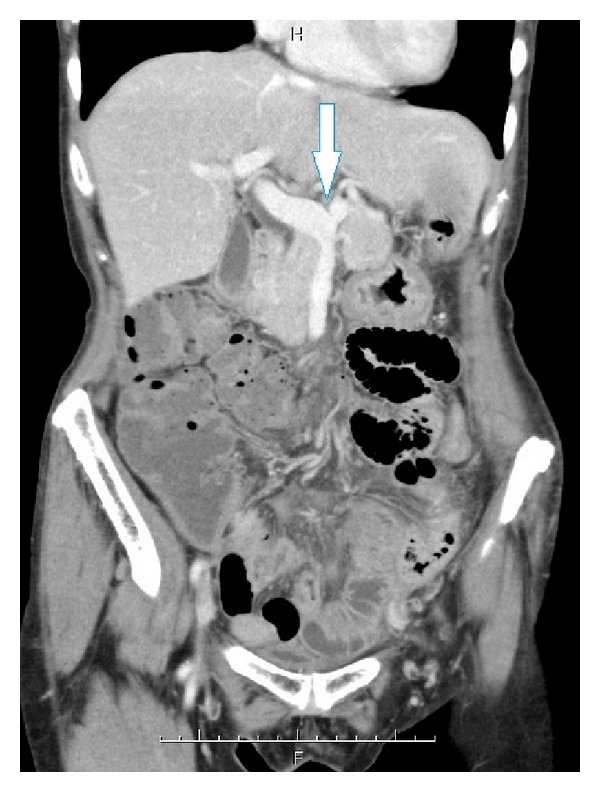
Contrast enhanced computed tomographic scan of the abdomen (coronal view) six days after starting anticoagulation therapy. The superior mesenteric vein is patent (arrow) and the thrombus has resolved.

The postoperative course was uneventful except for persistent anorexia. Ten days after the second exploration, CT scan was obtained due to the persistent anorexia. The CT scan revealed edematous small intestine and dilatation of the mesenteric veins. The scan also revealed a filling defect in the superior mesenteric vein (Figure 1) suggestive of a thrombus. She had no evidence of intestinal gangrene or peritonitis, and systemic heparin was begun followed by warfarin therapy. Laboratory data were not consistent with protein C, protein S, or antithrombin III deficiencies. She had no past history or family history of deep venous thrombosis or other coagulation disorders. Over the next six days, the thrombus resolved on repeat imaging studies (Figure 2), and her appetite recovered. She was discharged without further complications, continuing oral anticoagulation with warfarin.

## 3. Discussion

Mesenteric venous thrombosis is a rare cause of intestinal ischemia which rarely occurs after abdominal surgery. Its frequency remains obscure but Kim et al. reported that 0.3% of patients after laparoscopic bariatric surgery develop portomesenteric venous thrombosis [4]. James et al. reviewed 18 cases of portomesenteric venous thrombosis after laparoscopic procedures including Roux-en-Y gastric bypass, Nissen fundoplication, partial colectomy, cholecystectomy, and appendectomy [3]. To the best of our knowledge, this is the first reported case of superior mesenteric venous thrombosis after surgery for abdominal sepsis. Intra-abdominal inflammation such as acute pancreatitis can lead to mesenteric venous thrombosis [1, 3, 5, 6]. In the present patient, we believe that inflammation due to intestinal perforation led to superior mesenteric venous thrombosis. While the exact etiology of this complication is unclear, the use of the laparoscope in the first operation may also have contributed to this complication.

The etiologic factors associated with mesenteric venous thrombosis are varied, most commonly associated with coagulation disorders such as Factor V Leiden, protein C and protein S deficiencies, and antithrombin III deficiency [1–3, 5, 6]. In young women, oral-contraceptive use can lead to mesenteric venous thrombosis [1, 2, 6]. Other etiologic factors include inflammatory conditions such as pancreatitis, intra-abdominal sepsis, cirrhosis, portal hypertension, neoplasms, and blunt abdominal trauma [1, 2, 5, 6].

The diagnosis of mesenteric venous thrombosis is often delayed because the associated symptoms are usually subtle or nonspecific. The most common symptom is unexplained abdominal pain. Abdominal distension, anorexia, diarrhea, and vomiting have also been associated with mesenteric venous thrombosis [1–3, 5]. Severe abdominal pain, fever, and peritoneal signs suggest intestinal infarction or perforation. In the present patient, she had only unexplained anorexia which resolved after treatment. CT scan is considered to be the best way to establish the diagnosis of mesenteric venous thrombosis, with a sensitivity as high as 90% [1, 2]. In patients with mesenteric venous thrombosis, a central lucency in the mesenteric vein may be seen on CT scan. Other CT findings include enlargement of the superior mesenteric vein, a sharply defined vein wall with a rim of increased density, and intestinal edema [1].

The treatment of mesenteric venous thrombosis includes systemic anticoagulation. Surgical intervention is sometimes required. When the diagnosis of mesenteric venous thrombosis is made, systemic anticoagulation with heparin should be started immediately [1, 2]. Brunaud et al. reported that, in patients without bowel necrosis or perforation, the morbidity, mortality, and survival rate are similar to both surgical and nonoperative management [2]. However, in patients with intestinal infarction, peritonitis, or bowel stricture due to ischemia, surgery is essential. Warfarin should be started in the absence of intestinal ischemia [1]. Nasogastric suction, fluid resuscitation, and bowel rest are included as supportive care [1, 4].

This rare complication must be considered when evaluating patients after abdominal surgery with unexplained abdominal symptoms. Nonoperative management is feasible when indicated.
